# Special Issue: Microbial Biofilms in Healthcare: Formation, Prevention and Treatment

**DOI:** 10.3390/ma12122001

**Published:** 2019-06-22

**Authors:** Karen Vickery

**Affiliations:** Surgical Infection Research Group, Faculty of Medicine and Health Sciences, Macquarie University, Sydney 2109, Australia; karen.vickery@mq.edu.au

**Keywords:** biofilms, dry surface biofilms, periodontitis, breast implants, *Candida auris*, calcium sulphate, antibiotic, topical negative pressure wound therapy, antimicrobial peptides

## Abstract

Biofilms are a structured community of microorganisms that are attached to a surface. Individual bacteria are embedded in a bacterial-secreted matrix. Biofilms have significantly increased tolerance to removal by cleaning agents and killing by disinfectants and antibiotics. This special issue is devoted to diagnosis and treatment of biofilm-related diseases in man. It highlights the differences between the biofilm and planktonic (single cell) lifestyles and the diseases biofilms cause from periodontitis to breast implant capsular contracture. Biofilm-specific treatment options are detailed in experimental and review manuscripts.

## Introduction

Biofilms are ubiquitous with an estimated 99% of the world’s bacteria living enclosed in a biofilm. The problems that biofilm cause in industry have been well documented and methods to reduce their impact have been explored since before the middle of the last century. However, the extent to which biofilms play a significant detrimental role in chronic disease and implantable medical device failure has only been acknowledged over the past few decades whilst the role they play in surface and surgical instrument decontamination failure has only recently been highlighted.

Biofilms are a structured community of microorganisms that are attached to a surface. In healthcare, environmental biofilms take three forms: traditional hydrated biofilms which form in wet areas such as showers, water pipes and sinks; biofilms that form on dry surfaces such as benchtops and curtains, called dry surface biofilms (DSB); and build-up biofilms (BUB) that form on surgical instruments subjected to cycles of use, decontamination (cleaning and disinfection) and drying during storage. In addition, biofilm forms in human tissue such as the lung of cystic fibrosis sufferers and in chronic wounds, and biofilms on implantable medical devices lead to their failure. The importance of biofilms in healthcare arises due to biofilms’ increased tolerance to biocides and increased tolerance to desiccation when compared with planktonic organisms of the same species.

Biofilms’ increased tolerance to desiccation means that they can survive dry conditions which readily kills planktonic bacteria. DSB have been shown to survive over 12 months in a sterile container, on a bench without any nutrition, and they are particularly tolerant to disinfectants [1,2]. DSB have been detected on over 90% of dry hospital surfaces in four countries (Australia, Brazil, Saudi Arabia and the United Kingdom) [1,3,4,5]. In this special issue, Ledwoch and Maillard investigated the efficacy of 12 commercial disinfectants and 1000 ppm sodium hypochlorite (recommended as the disinfectant of choice by Public Health England) against DSB composed of *Candida auris* [6]. They initially developed a DSB model of this emerging pathogen and then used this model DSB in a modification of the ASTM2967-15 Wiperator test to measure decrease in *C. auris* viability, transfer of *C. auris* and biofilm re-growth following treatment. Similar to bacterial DSB, *C. auris* DSB showed increased tolerance to common disinfectant agents.

Bacteria can attach to host tissue and any implantable medical device. In this issue, Kamaruzzaman et al. review the bacterial species that are principally isolated from healthcare associated infection, the body sites where biofilms cause disease, diagnosis and treatment options [7]. They go on to describe the mechanisms of antimicrobial tolerance and evasion of host immune response which biofilm exhibits. Mempin et al. review the surface characteristics of different types of breast implants and how this affects bacterial attachment [8]. They also describe how biofilm formation on breast implants leads to capsular contracture and its possible role in the potentiation of breast implant associated Anaplastic Large Cell Lymphoma (ALCL). Frédéric et al. review the role that oral biofilm plays in periodontitis and peri-implantitis and the limitations of treatment options [9]. The poor response of chronic wounds to treatment promoted Tahir et al. to investigate whether physically altering biofilms’ architecture increased its sensitivity to biocides [10]. They did this by utilizing their topical negative pressure wound therapy model. In this special issue, other treatment options that were experimentally explored included Laycock et al.’s work on the efficacy of antibiotic release from calcium sulphate bone void filler beads [11]. As calcium sulphate is completely biocompatible and absorbed by the body, combining it with antibiotics and using this combination locally would serve to increase antibiotic release at fracture sites and reduce the need for high dose systemic use of antibiotics.

In this special issue, three reviews address various antibiofilm treatment strategies. Biofilm formation and maturation can be stopped by preventing bacterial attachment or by interfering with bacterial quorum sensing. Once formed, biofilm removal can be induced by use of chemical and quorum sensing dispersal agents. Beitelshees et al. reviewed biofilm formation and how bacterial phenotype changes during biofilm development [12]. They relate bacterial phenotype to the anti-biofilm strategy. Yasir et al. reviewed the major antibiofilm mechanisms of the action of antimicrobial peptides and how these prevent biofilm formation and disrupt mature biofilms [13]. Subhadra et al. reviewed the recent advances in preventing biofilm formation and inducing its dispersal by interfering with quorum sensing [14].

## References

[1] Hu H., Johani K., Gosbell I.B., Jacombs A., Almatroudi A., Whiteley G.S., Deva A.K., Jensen S., Vickery K. (2015). Intensive care unit environmental surfaces are contaminated by multiresistant bacteria in biofilms: Combined results of conventional culture, pyrosequencing, scanning electron microscopy and confocal laser microscopy. J. Hosp. Infect..

[2] Almatroudi A., Gosbell I., Hu H., Jensen S., Espedido B., Tahir S., Glasbey T., Legge P., Whiteley G., Deva A. (2016). Staphylococcus aureus dry-surface biofilms are not killed by sodium hypochlorite: Implications for infection control. J. Hosp. Infect..

[3] Costa D., Johani K., Melo D.S., Lopes L., Lima L.L., Tipple A., Hu H., Vickery K., Costa D.D.M., Lopes L.K.D.O. (2019). Biofilm contamination of high-touched surfaces in intensive care units: Epidemiology and potential impacts. Lett. Appl. Microbiol..

[4] Johani K., Abualsaud D., Costa D.M., Hu H., Whiteley G., Deva A., Vickery K. (2018). Characterization of microbial community composition, antimicrobial resistance and biofilm on intensive care surfaces. J. Infect. Public Health.

[5] Ledwoch K., Dancer S., Otter J., Kerr K., Roposte D., Rushton L., Weiser R., Mahenthiralingam E., Muir D., Maillard J.-Y. (2018). Beware biofilm! Dry biofilms containing bacterial pathogens on multiple healthcare surfaces; a multi-centre study. J. Hosp. Infect..

[6] Ledwoch K., Maillard J.-Y. (2018). Candida auris Dry Surface Biofilm (DSB) for Disinfectant Efficacy Testing. Materials.

[7] Kamaruzzaman N.F., Tan L.P., Yazid K.A.M., Saeed S.I., Hamdan R.H., Choong S.S., Wong W.K., Chivu A., Gibson A.J., Yazid K.M. (2018). Targeting the Bacterial Protective Armour; Challenges and Novel Strategies in the Treatment of Microbial Biofilm. Materials.

[8] Mempin M., Hu H., Chowdhury D., Deva A., Vickery K. (2018). The A, B and C’s of Silicone Breast Implants: Anaplastic Large Cell Lymphoma, Biofilm and Capsular Contracture. Materials.

[9] Lasserre J.F., Brecx M.C., Toma S., Frédéric L.J., Michel B., Selena T. (2018). Oral Microbes, Biofilms and Their Role in Periodontal and Peri-Implant Diseases. Materials.

[10] Tahir S., Malone M., Hu H., Deva A., Vickery K. (2018). The Effect of Negative Pressure Wound Therapy with and without Instillation on Mature Biofilms In Vitro. Materials.

[11] Laycock P.A., Cooper J.J., Howlin R.P., Delury C., Aiken S., Stoodley P. (2018). In Vitro Efficacy of Antibiotics Released from Calcium Sulfate Bone Void Filler Beads. Materials.

[12] Beitelshees M., Hill A., Jones C.H., Pfeifer B.A. (2018). Phenotypic Variation during Biofilm Formation: Implications for Anti-Biofilm Therapeutic Design. Materials.

[13] Yasir M., Willcox M.D.P., Dutta D. (2018). Action of Antimicrobial Peptides against Bacterial Biofilms. Materials.

[14] Subhadra B., Kim D.H., Woo K., Surendran S., Choi C.H. (2018). Control of Biofilm Formation in Healthcare: Recent Advances Exploiting Quorum-Sensing Interference Strategies and Multidrug Efflux Pump Inhibitors. Materials.

